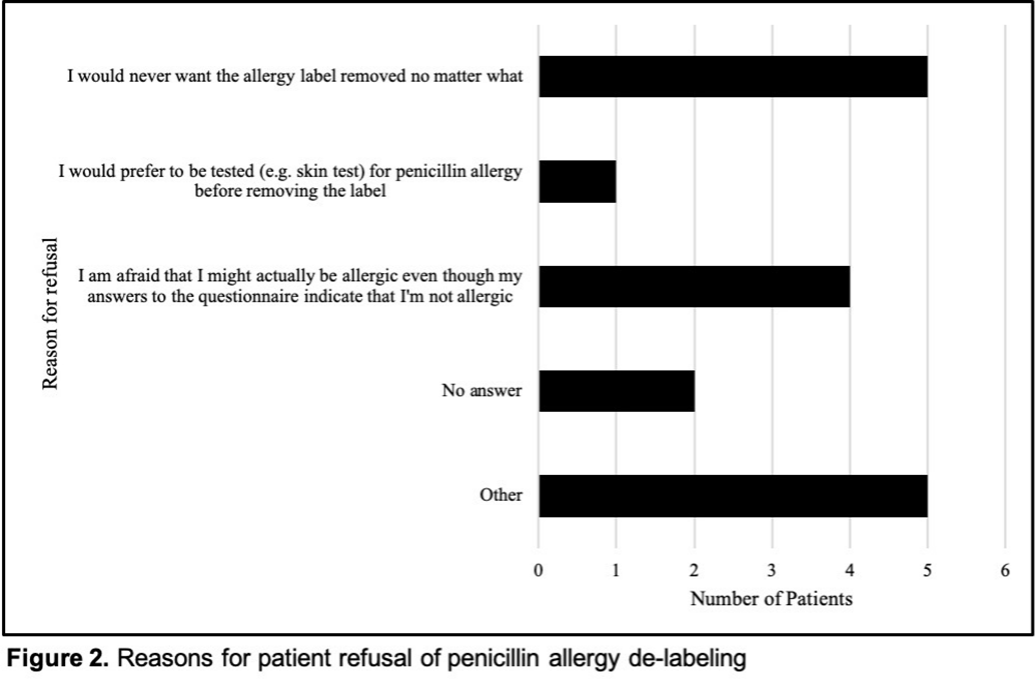# Impact of an Inpatient Nurse-Initiated Penicillin Allergy Delabeling Questionnaire

**DOI:** 10.1017/ash.2021.71

**Published:** 2021-07-29

**Authors:** Lauren Dutcher, Hilary Bediako, Christina Harker, Aditi Rao, Kristen Sigafus, Keith Hamilton, Olajumoke Fadugba

## Abstract

**Background:** Penicillin allergy is the most common drug allergy, with ~10% of all patients in the United States reporting a penicillin allergy. A penicillin allergy label is associated with the use of inappropriate or broad-spectrum antibiotics, worse patient outcomes, increased bacterial resistance, and increased healthcare costs, yet no studies have explored the unique role nurses may play in allergy delabeling through history taking as a part of broader antimicrobial stewardship efforts. Here, we describe the impact of using an inpatient nurse-initiated penicillin-allergy questionnaire. **Methods:** We implemented a nurse-driven intervention focused on penicillin allergy delabeling in inpatient noncritical care units (surgery, neurology, medicine, oncology, and cardiovascular medicine) at an academic hospital from July 9, 2019, to July 24, 2020. Patients with a penicillin allergy listed in the electronic health record (EHR) were identified and invited to participate. The intervention consisted of a questionnaire administered by nurses who elicited details of penicillin allergy history. If a patient was deemed eligible for penicillin allergy removal, nurses requested approval from both the patient as well as a physician member of the study team. **Results:** In total, 306 patients with a penicillin allergy label were identified in the EHR, of whom 242 patients were eligible for and agreed to participate in the delabeling interview (Figure 1). Of the 34 (14%) of 242 patients potentially eligible for delabeling by the questionnaire based on their history, the study physicians agreed with delabeling for 23 (68%) of 34 patients. Of these 34 patients, 18 (53%) agreed with delabeling (pending physician approval), and 16 (47%) of these 34 patients were ultimately delabeled. For those who declined delabeling, never wanting the label removed under any circumstance and uncertainty about accuracy of the survey results were common reasons for refusal (Figure 2). Additionally, for the 13 patients who refused delabeling, 9 patients did not want or were unsure about following up with an allergy specialist. **Conclusions:** The nurse-driven penicillin-allergy delabeling questionnaire is a no-cost intervention that can successfully identify patients to delabel. In this study, this measure resulted in the removal of 16 (7%) of 242 penicillin allergy labels. However, patients frequently opted to keep penicillin allergy labels, expressing uncertainty and fear of removal. Future work should explore optimal methods to engage nurses and patients in allergy delabeling, as well as the impact on antibiotic use and patient outcomes.

**Funding:** No

**Disclosures:** None

**Figure 1.**
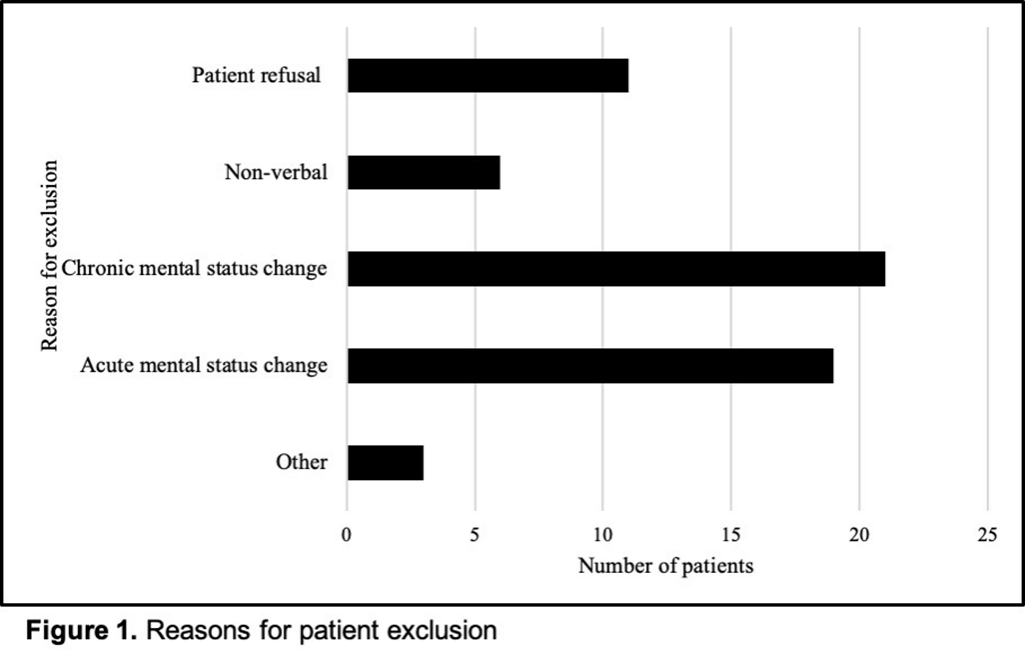

**Figure 2.**